# Cloning and Functional Analysis of FLJ20420: A Novel Transcription Factor for the BAG-1 Promoter

**DOI:** 10.1371/journal.pone.0034832

**Published:** 2012-05-02

**Authors:** Hongyu Liu, Ying Li, Yongwen Li, Baoxin Liu, Heng Wu, Jing Wang, Yuli Wang, Min Wang, Shou-Ching Tang, Qinghua Zhou, Jun Chen

**Affiliations:** 1 Tianjin Key Laboratory of Lung Cancer Metastasis and Tumor Microenvironment, Tianjin Lung Cancer Institute, Tianjin Medical University General Hospital, Heping District, Tianjin, China; 2 The Department of Thoracic Surgery, Henan Tumor Hospital, Zhengzhou, Henan, China; 3 Division of Hematology/Oncology, Faculty of Medicine, University of Minnesota, Minneapolis, Minnesota, United States of America; 4 Tianjin Medical University Cancer Institute and Hospital, Hexi District, Tianjin, China; Karlsruhe Institute of Technology, Germany

## Abstract

BAG-1 is an anti-apoptotic protein that interacts with a variety of cellular molecules to inhibit apoptosis. The mechanisms by which BAG-1 interacts with other proteins to inhibit apoptosis have been extensively explored. However, it is currently unknown how BAG-1 expression is regulated at the molecular level, especially in cancer cells. Here we reported to clone a novel down-regulated BAG-1 expression gene named FLJ20420 using hBAG-1 promoter as a probe to screen Human Hela 5′ cDNA library by Southernwestern blot. The FLJ20420 gene encodes a ∼26-kDa protein that is localized in both the cytoplasm and nucleus. We proved that FLJ20420 protein can specially bind hBAG-1 promoter region by EMSA in vivo and ChIP assay in vivo. Northern blot analysis revealed a low level of FLJ20420 transcriptional expression in normal human tissues (i.e., brain, placenta, lung, liver, kidney, pancreas and cervix), except for heart and skeletal muscles, which showed higher levels. Furthermore, enhanced FLJ20420 expression was observed in tumor cell lines (i.e., MDA468, BT-20, MCF-7, C33A, HeLa and Caski). Knockdown of endogenous FLJ20420 expression significantly increased BAG-1 expression in A549 and L9981 cells, and also significantly enhanced their sensitivity to cisplatin-induced apoptosis. A microarray assay of the FLJ20420 siRNA –transfectants showed altered expression of 505 known genes, including 272 upregulated and 233 downregulated genes. Finally, our gene array studies in lung cancer tissue samples revealed a significant increase in FLJ20420 expression in primary lung cancer relative to the paired normal lung tissue controls (p = 0.0006). The increased expression of FLJ20420 corresponded to a significant decrease in BAG-1 protein expression in the primary lung cancers, relative to the paired normal lung tissue controls (p = 0.0001). Taken together, our experiments suggest that FLJ20420 functions as a down-regulator of BAG-1 expression. Its abnormal expression may be involved in the oncogenesis of human malignancies such as lung cancer.

## Introduction

BAG-1 is a multifunctional protein that plays important roles in apoptosis, cell survival, transcription, cell motility and proliferation. In addition, BAG-1 expression is often altered in various human malignancies, especially in human breast cancer, lung cancer and cervical cancer [1], [2]. Furthermore, BAG-1 expression has been associated with the prognosis of a variety of human malignancies, such as breast cancer and lung cancer [3], [4], [5].

It is thought that the pleiotropic effects of BAG-1 are due to its interaction with diverse cellular targets. The proteins Hsc70, Hsp70, Bcl-2 and RAF-1 kinase, as well as nuclear hormone receptors and subunits of the ubiquitination-proteasome system, are all known BAG-1- interacting partners. BAG-1 interacts with Hsp70 via a C-terminal BAG domain which allows it to facilitate the nucleotide exchange [6], [7]. Many functions of BAG-1 in cell apoptosis and cell survival are dependent on the BAG domain. Given the role of BAG-1 in a number of different biological pathways, it is not surprising that deregulated BAG-1 expression is associated with tumorigenesis.

BAG-1 is expressed as three major isoforms, designated as p50 (BAG-1L), p46 (BAG-1M), and p33 (BAG-1S), as well as one minor isoform, p29. The apparent molecular masses of the major and minor isoforms are 50, 46, 33 and 29 kDa, respectively. Different BAG-1 isoforms have different biological functions in different cancer cell lines and tissues [8], [9]. We have provided convincing *in vitro* evidence that the four protein products are generated from a single mRNA and translated by alternative initiation from the four different start codons through a leaky scanning mechanism [10]. Willis and co-workers also demonstrated that, *in vivo,* BAG-1S synthesis is dependent on the presence of an internal ribosome entry segment (IRES) in the 5′-UTR of BAG-1 mRNA. Their studies also showed that the polypyrimidine tract binding protein 1 (PTB-1) and poly (rC) binding protein 1 (PCBP1) stimulate IRES-mediated translation initiation [11]. Furthermore, Willis and co-workers also reported that BAG-1 IRES activity was promoted by structural changes mediated by the PCBP1 and PTB-1 complex [12]. However, the molecules that regulate BAG-1 transcription, especially in malignant cells, have not been well studied.

We previously isolated and characterized the human BAG-1 promoter of an 890-bp DNA fragment in the 5′ region [13], which allowed us to study the transcriptional control of BAG-1. Since BAG-1 is over-expressed in the human cervical cancer cell line HeLa, we attempted to identify proteins that bind to the BAG-1 promoter by Southwestern blot analysis, using the BAG-1 promoter as a probe to screen the Human HeLa 5′ stretch plus cDNA library, λTripIEx (Clontech). In this study we describe the molecular cloning and functional characterization of a cDNA that encodes a novel protein that physically binds to the BAG-1 promoter between −483 and –433 bp. Sequence comparison in the human genome bank identified the gene as FLJ20420, which has no known function. Functional analysis of FLJ20420 revealed that this protein downregulates BAG-1 expression in lung cancer cell lines, suggesting that it may play a role in the regulation of BAG-1 expression in human carcinogenesis.

## Results

### Molecular Cloning of FLJ20420

We used the BAG-1 promoter region of an 890-bp DNA as a probe to screen the HeLa cDNA library, λTripIEx, by Southwestern blot analysis. After screening more than 5×10^6^ plaques, we obtained two tertiary positive clones. A search of the NCBI database revealed that one of the clones was identical to the *Homo sapiens* cDNA termed FLJ20420 (GI: 7020507). The FLJ20420 gene encodes an uncharacterized coiled-coil-helix-coiled-coil-helix domain containing protein 3 (Chchd3), which shares approximately 90% sequence homology with the mouse Chchd3 protein (GI: 62510510), 85% homology with rat Chchd3 (GI: 62646993) and 99% homology with chimpanzee Chchd3 (GI: 55629442). The DNA and protein sequences are shown in Figure S1. This positive clone contains the complete 227-amino acid open reading frame. Next, we checked the FLJ20420 sequence in the human genome database and determined that it is located on chromosome 7, and has 8 small exons.

### In vitro Binding of FLJ20420 Protein to BAG-1 Promoter

The apparent molecular weight of FLJ20420 and GST-FLJ20420 proteins was determined to be ∼26 kDa and ∼50 kDa, respectively (Figure S2a). The HisC-FLJ20420 fusion protein was also translated *in vitro* using the TNT Quick Translation kit with linearized pcDNA3.1/HisC-FLJ20420 plasmid. Protein expression was confirmed by Western blotting using an anti-His antibody, which demonstrated an apparent molecular weight of ∼30 kDa (Figure S2b).

**Figure 1:**
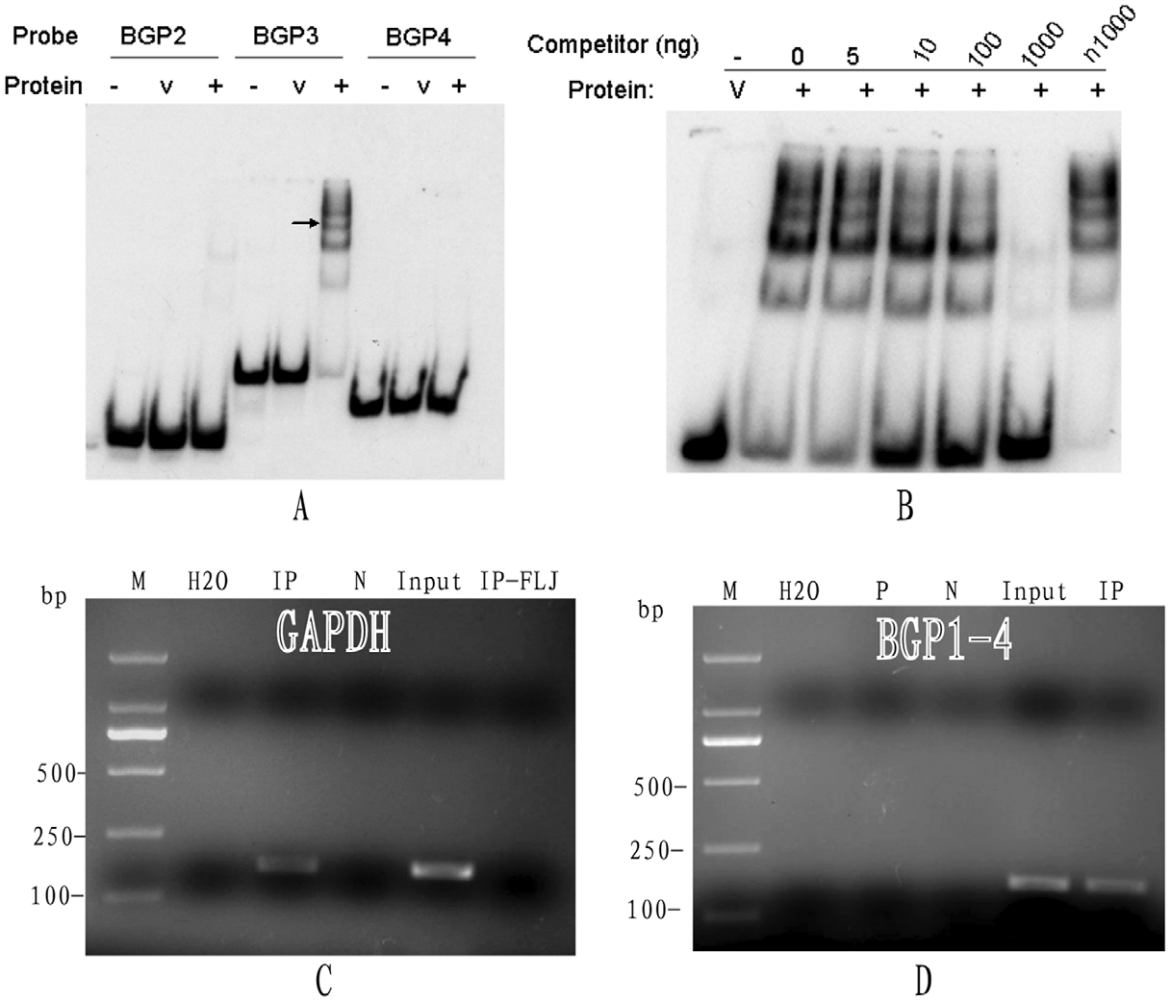
The binding assays of FLJ20420 to the BAG-1 promoter. (A) The BAG-1 promoter was separated into several 30- to 50-bp DNA oligos, which were used as probes to perform EMSAs with purified GST-FLJ20420 fusion protein (+). Purified GST protein was used as a control (v). (B) EMSA competition test. Unlabeled specific DNA fragments (i.e., 100 ng and 1000 ng), and not the non-specific fragment (i.e., n1000) effectively compete for FLJ20420 binding. (C) Immunoprecipitation of crosslinked protein/DNA with anti-acetyl histone H3 antibody was first performed, and then purified DNA was analyzed by PCR using the control primers (GAPDH). M: DNA ladder; H_2_O: water; IP: immunoprecipitation with anti-acetyl histone H3 antibody; N: negative control, immunoprecipitation with normal rabbit IgG antibody; Input: mixed DNA; IP-FLJ: immunoprecipitation with anti-human FLJ20420 antibody. (D) Immunoprecipitation of crosslinked protein/DNA with anti-human FLJ20402 antibody was first performed, and then purified DNA was analyzed by PCR using BAG-1 promoter-specific primers (BGP1-4). M: DNA ladder; H_2_O: water; P: immunoprecipitation with anti-acetyl histone H3 antibody; N: negative control, immunoprecipitation with normal rabbit IgG antibody; IP: immunoprecipitation with anti-human FLJ20420 antibody; Input: mixed DNA.

We next determined whether the FLJ20420 protein specifically binds to the BAG-1 promoter *in vitro*. To this end, the full length BAG-1 promoter was first separated into several smaller DNA fragments of sizes ranging between 150 and 230 bp. These fragments were used in a gel shift assay with the purified GST-FLJ20420 fusion protein. The positive DNA fragment was separated once again into oligos between 30 and 50 bp in length. As shown in Figure 1A, GST-FLJ20420 bound to the BGP3 DNA fragment (−483 to –433 bp). Furthermore, a competition binding test also demonstrated that the FLJ20420 protein specifically bound to the BGP3 region (Figure 1B). These results confirmed the specific *in vitro* binding of the FLJ20420 protein to the BAG promoter. A ChIP assay was then performed to determine whether FLJ20420 protein can specifically bind to the BAG-1 promoter in vivo. We first performed immunoprecipitation of crosslinked protein/DNA with anti-human FLJ20420 antibody, and then purified DNA was analyzed by PCR using BAG-1 promoter-specific primers. GAPDH was used as a control for the system (Figure 1C). As shown in Figure 1D, the PCR product was observed in the primer of the BAG promoter (BGP1-4), which amplified a 175-bp DNA fragment at −513∼−338 bp upstream the BAG-1 promoter region. This region includes the physically binding region of the BGP3 DNA fragment (−483 to –433 bp) in vitro, and indicates that FLJ20420 protein directly binds to the BAG-1 promoter in vivo. This PCR product was also confirmed by sequencing to be the BAG-1 promoter.

### FLJ20420 Expression in Normal Tissues and Tumor Cell Lines

**Figure 2:**
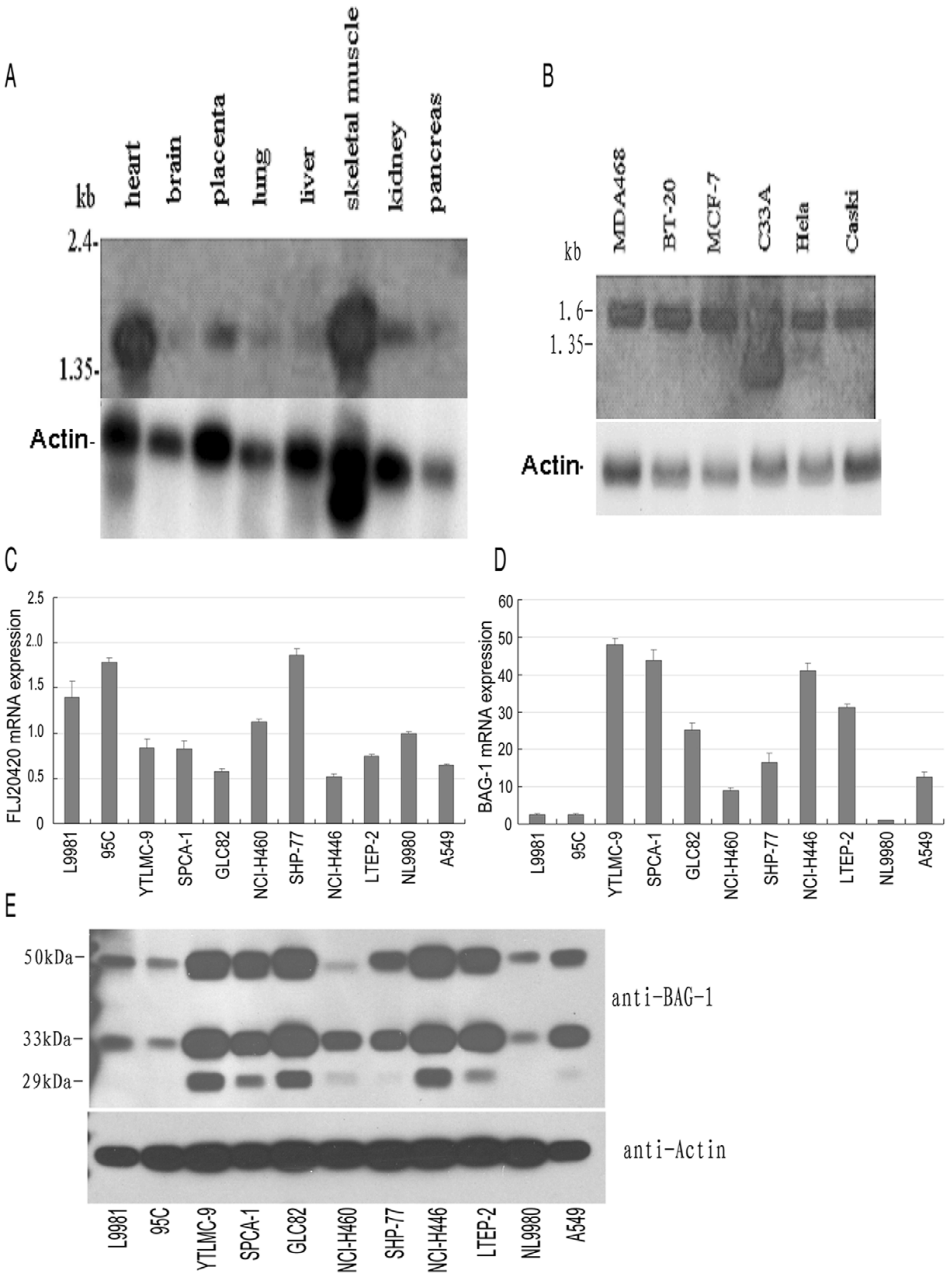
FLJ20420 mRNA expression in different tissues and cell lines. Northern blot analysis of FLJ20420 mRNA expression in human normal tissues (A) and various tumor cell lines (B). FLJ20420 cDNA was used as a probe. The number on the left side indicates the size of the molecular mass markers. The same blot was then stripped and re-probed with human actin probe as an internal control. (C-D) Real-time PCR analysis of FLJ20420 and BAG-1 mRNA expression in lung cancer cell lines. NL9980 cells were used as a calibrator. The expression of FLJ20420 opposed BAG-1 expression in these cell lines. Error bars are representative of an average of 2 triplicate RT-PCR experiments. (E) Immunoblot analysis shows that BAG-1 protein expression corresponds to BAG-1 mRNA expression in the cell lines analyzed.

To determine whether FLJ20420 is expressed in normal tissues, a Human MTN RNA Blot (Clontech) was used to assess FLJ20420 RNA expression. As shown in Figure 2A, FLJ20420 RNA was present in most normal tissues, including heart, skeletal, brain, placenta, lung, liver, kidney and pancreas. However, increased expression levels were observed in heart and skeletal muscles (Figure 2A). Furthermore, MTC multiple human cell line cDNA panels, which included breast cancer cells (MDA468, BT-20 and MCF-7), cervical cancer cells (C33A and HeLa) and lung cancer cells (Caski), also exhibited increased FLJ20420 expression levels. Interestingly, the C33A cell line possessed two FLJ20420 bands. One of the bands corresponded to the same band size observed in the other cancer cell lines, while the second band appeared to be of smaller size (Figure 2B).

Next, the expression of FLJ20420 and BAG-1 in human lung cancer cell lines was investigated. There were eleven human lung cancer cell lines studied, which included five adenocarcinoma cell lines (A549, GLC82, LTEP-2, SPCA-1 and 95C), one squamous carcinoma cell line (YTLMC-9), three large cell lung cancer cell lines (NCI-H460, NL9980 and L9981) and two small cell lung cancer cell lines (SHP-77 and NCI-H446). As shown in Figure 2C, relative to the other cell lines, the human lung cancer cell lines NL9980, SHP-77, NCI-H460, 95C and L9981 all exhibited a higher level of FLJ20420 expression, as determined by real-time PCR. Conversely, these same cell lines (NL9980, SHP-77, NCI-H460, 95C and L9981) also demonstrated lower levels of BAG-1 expression compared to higher BAG-1 expression levels in the remaining cell lines (GLC82, LTEP-2, NCI-H446, SPCA-1 and YTLMC-9 ) (Figure 2D). The mRNA expression levels of FLJ20420 and BAG-1 were determined to be inversely related based on Pearson’s chi-square test. (χ^2^ = 0.681, p = 0.000). Furthermore, Western blot analysis was also used to investigate BAG-1 protein expression in all of the lung cancer cell lines. Similarly, a higher level of BAG-1 protein was observed in the lung cancer cell lines GLC82, LTEP-2, SPCA-1, NCI-H446, YTLMC-9 and A549 (Figure 2E), which corresponded to increased BAG-1 mRNA expression.

### FLJ20420 Decreased BAG-1 Expression in Lung Cancer Cell Lines A549 and L9981

**Figure 3:**
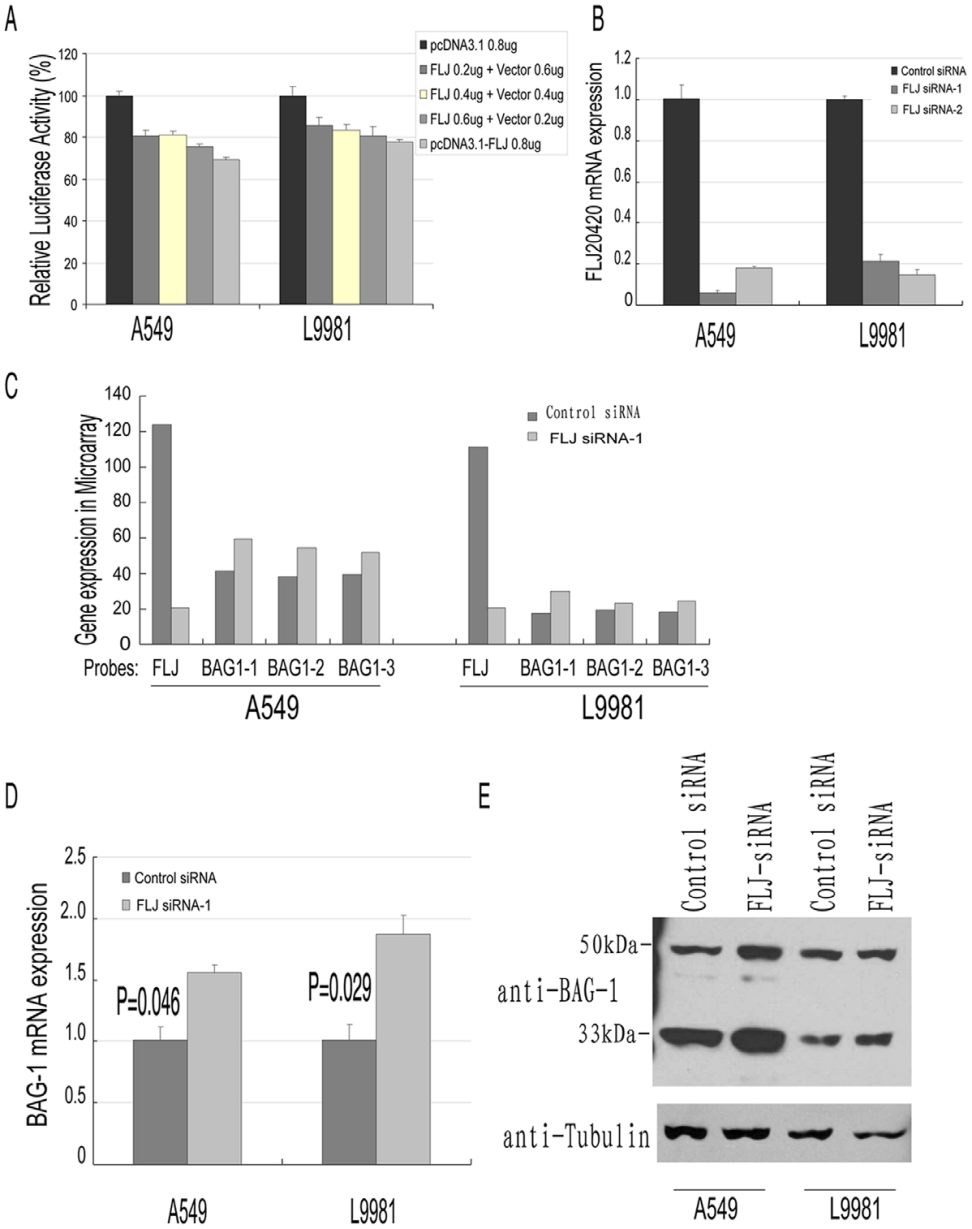
FLJ20420 down-regulates the expression of BAG-1 in lung cancer cell lines. (A) Luciferase assay with BAG-1 promoter. Luciferase reporter under the control of the BAG-1 promoter was co-transfected in A549 and L9981 cells with either the pcDNA3.1 parent vector or pcDNA3.1-FLJ20420 expression plasmid. Luciferase activity for the BAG-1 promoter is set as 100% upon transfection with the empty pcDNA3.1 vector. Transfection efficiency was determined by co-transfecting the pRL-CMV vector, which encoded the Renilla luciferase gene (0.02 µg). (B) Knockdown of FLJ20420 expression in A549 and L9981 cell lines. Cells were transfected with either FLJ20420 siRNA-1, -2 or scrambled control siRNA. After 48 h of transfection, the cells were collected and total RNA was extracted. FLJ20420 mRNA expression was measured by real-time PCR, in triplicate, as described in the methods section. (C) Microarrays of FLJ20420-silenced A549 and L9981 cells. In both of the FLJ20420-silenced A549 and L9981 cell lines, FLJ20420 expression was dramatically reduced, while BAG-1 expression increased, in comparison to control transfected cells. (D) BAG-1 mRNA expression in FLJ204200-siRNA transfected cells, as measured in triplicate by real-time PCR, and described in the methods section. (E) BAG-1 protein expression in FLJ20420-siRNA transfected cells was detected by Western blotting with anti-BAG-1 antibody at the same time points post-transfection. Tubulin was used as an internal control.

**Figure 4:**
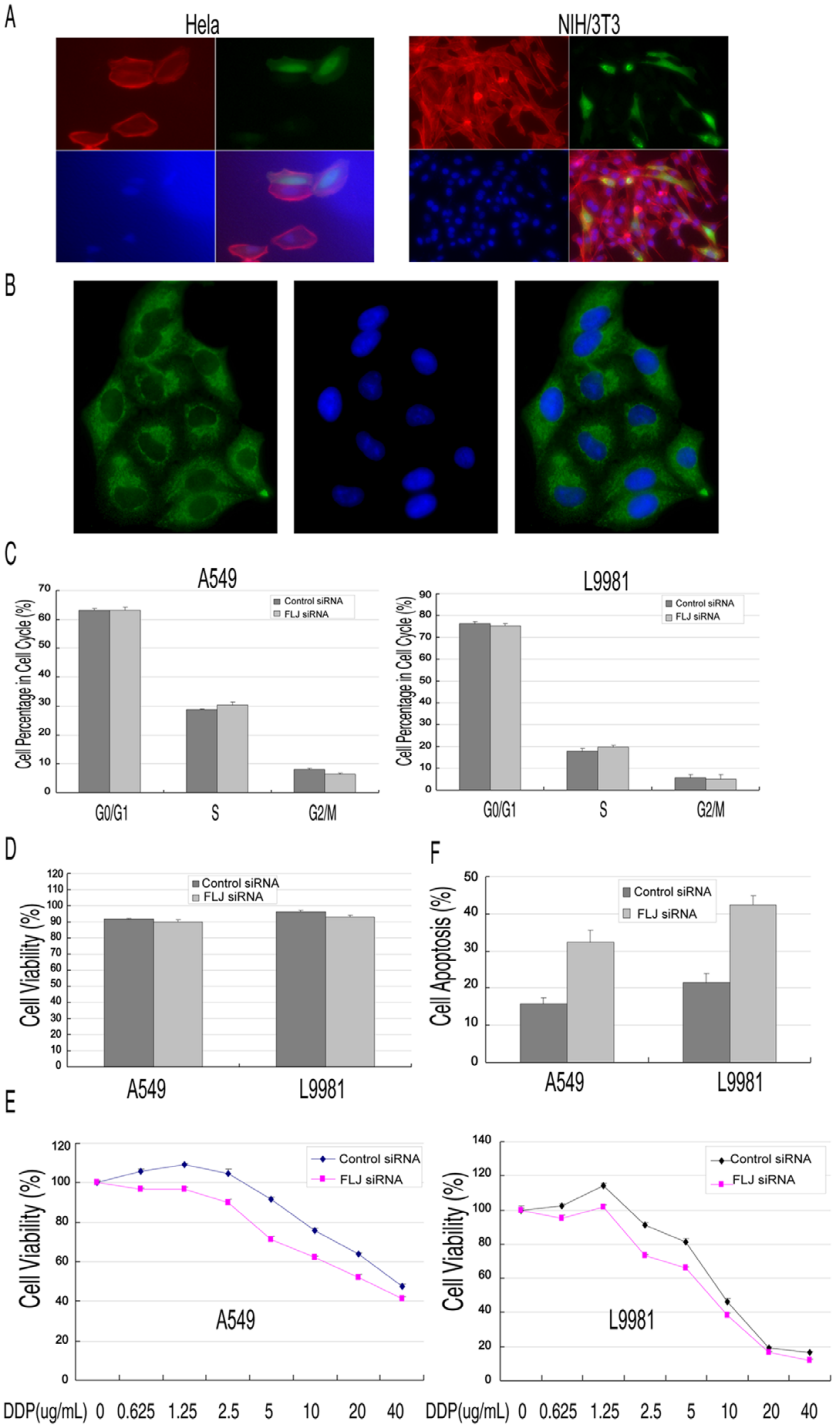
Function of the FLJ20420 gene. (A–B) The subcellular localization of FLJ20420 protein. (A) Cells were transiently transfected with pEF1-HisC-FLJ20420 for 48 h, fixed with 4% paraformaldehyde, and analyzed by immunofluorescence. Monoclonal anti-Xpress^TM^ antibody (Invitrogen, CA), at a dilution of 1∶500, was used to detect the FLJ20420 fusion protein (green). Phalloidin (Sigma), at a dilution of 1∶500, was used to stain the cell skeleton (red). DAPI (Sigma), at a dilution of 1∶5000, was used to stain cell nuclei (blue). FLJ20420 protein expression is apparent in both the cytoplasm and nuclei of HeLa cells and NIH/3T3 cells. (B) A549 cells were detected by immunofluorescence with monoclonal anti-FLJ20420 antibody at a dilution of 1∶1000 to detect the FLJ20420 native protein (green). DAPI (Sigma), at a dilution of 1∶5000, was used to stain cell nuclei (blue). FLJ20420 protein expression is apparent in both the cytoplasm and nuclei of A549 cells, but most in the cytoplasm. (C) Cell cycle analysis was evaluated by FACS after using PI to stain the cellular DNA. (D) Cell viability assay: 2×10^5^ cells/well were seeded in 6-well plates and transfected with specific or control siRNA. After incubation for 48 h, cell viability was determined by the MTT assay. (E) Cells transfected with FLJ20420 siRNA were treated with cisplatin (0, 0.625, 1.25, 2.5, 5, 10, 20, or 40 µg/ml) for 24 h and then analyzed by the MTT assay. (F) Cells transfected with FLJ20420 siRNA were treated with cisplatin (5 µg/ml) for 24 h and then analyzed for apoptosis by FACS.

To characterize the function of FLJ20420, A549 and L9981 cells were co-transfected with pcDNA3.1/FLJ20420 and the BGP-Luc (BAG-1 promoter) reporter plasmid. Luciferase activity for the BGP-Luc (BAG-1 promoter) was determined to be 100% upon transfection of the empty pcDNA3.1 vector. As shown in Figure 3A, co-transfection of increasing amounts of pcDNA3.1-FLJ20420 resulted in progressive decreases in luciferase activity from the BGP-Luc-(BAG-1 promoter). Specifically, the luciferase activity of A549 cells was observed to decrease from 100% to 69.16%, while in L9981 cells, luciferase activity decreased from 100% to 77.85% (see Figure 4A for complete data set demonstrating progressive decrease). Transfection of 0.8 µg pcDNA3.1-FLJ20420 reduced luciferase activity by 30.84% in A549 cells (100% vs 69.16%, P = 0.007) and 22.15% in L9981 cells (100% vs 77.85%, P = 0.014). However, luciferase activity was not affected by a similar amount of FLJ20420 plasmid on the pGL3-control and pGL3-Basic vectors (data not shown). Taken together, these results suggest that expression of FLJ20420 specifically inhibited the BAG-1 promoter, *in vitro*.

Next, we designed two distinct FLJ20420 siRNA (siRNA-1, -2) to knockdown FLJ20420 mRNA and protein expression in the A549 and L9981 cell lines. At 48 h after transfection with siRNA-1 and siRNA-2, the transcriptional expression of the FLJ20420 gene was significantly blocked (Figure 3B). Therefore, microarrays were used to study FLJ20420 mRNA changes in FLJ-siRNA-transfected A549 and L9981 cells. As shown in Figure 3C, relative to the scramble control, the expression level of FLJ20420 in both FLJ-siRNA-1 transfected cell lines was dramatically decreased. Since the Genechips contained three probes for BAG-1, each probe was designated as BAG-1 probe-1 (202387_at), -2 (229720_at) and -3 (211475_s_at). BAG-1 expression was increased in both A549 and L9981 cells that were transfected with FLJ-siRNA-1, relative to cells transfected with scramble control siRNA. Real-time PCR analysis confirmed increased BAG-1 expression levels in A549 and L9981 cells transfected with FLJ-siRNA-1, compared to negative siRNA-transfected cells (1.56-fold increase in expression, P = 0.046; 1.87-fold increase in expression, P = 0.029, respectively) (Figure 3D). Western blotting was also used to confirm these results. BAG-1 protein expression was increased in cells transfected with FLJ-siRNA-1, compared to the negative control cells (Figure 3E). Taken together, these results demonstrated that the FLJ20420 gene can negatively regulate BAG-1 expression in A549 and L9981 cells.

### The Biological Function of FLJ20420

The possible role of FLJ20420 as a transcription factor was explored by first studying the subcellular localization of FLJ20420 in HeLa and NIH/3T3 cells by immunofluorescence. As shown in Figure 4A, FLJ20420 protein localized in both the cytoplasm and nucleus of the cell, which is consistent with its function as a BAG-1 transcription factor. Next, the subcellular localization of native FLJ20420 protein was also determined in A549 cells with monoclonal anti-FLJ20420 antibody (Figure 4B). The results showed that FLJ20420 protein localized in both the cytoplasm and nucleus, but mostly in the cytoplasm of A549 cells.

The biological function of FLJ20420 in cell cycle control and cell viability was also studied by analyzing cell cycle changes in A549 and L9981 cells transfected with FLJ-siRNA-1. As illustrated in Figure 4C, 48 h after transfection, 30.46±0.83% and 6.344±0.549% of FLJ-20420-silenced A549 cells were in S and G2/M phase, respectively. In contrast, 28.77±0.288% and 7.982±0.4007% of control cells were shown to be in S and G2/M phase, respectively (P>0.05). Similar results were also observed in FLJ20420-silenced L9981 cells compared to control cells (L9981 cells: 19.75±0.8905% and 5.07±0.264; control cells: 17.87±1.2% and 5.74±0.325%, in S and G2/M phase, P>0.05, respectively). There was no significant difference in cell viability between the control and FLJ20420-siRNA-transfected cells (A549∶91.505±0.544% vs 89.942±1.28%, P>0.05; L9981∶96.208±1.032% vs 93.049±1.269%, P>0.05, respectively) (Figure 4D). These results indicated that FLJ20420 may not directly affect the cell cycle. The role of FLJ20420 in apoptosis was evaluated in FLJ20420-siRNA-transfected A549 and NL9981 cells treated with various concentrations of cisplatin (DDP) (i.e., 0, 0.625, 1.25, 2.5, 5, 10, 20, 40 µg/mL). After 24 h of DDP exposure, cell viability was assessed by the MTT assay. The percent of cell viability following DDP treatment was calculated based on the total number of untreated cells, as shown in Figure 4E. Compared to the control siRNA-transfected cells, A549 and L9981 cells transfected with FLJ20420-siRNA showed enhanced sensitivity to DDP-induced apoptosis, especially at 2.5 and 5 µg/ml DDP (A549∶89.96% vs 104.91%, 71.77% vs 91.67%, p<0.05, respectively; L9981∶73.18% vs 91.12%, 65.99% vs 81.37%, p<0.05, respectively). We then determined the occurrence of apoptotic cells by Annexin-V FITC flow cytometry. The data were analyzed by first determining the percentage of apoptotic cells in the DDP-treated cell population and then subtracting the percentage of apoptotic cells in the DDP-untreated cell population. The percentage of apoptotic cells in the DDP-untreated cells, which were transfected with the control siRNA or FLJ-siRNA, showed no significant difference (all with less than 5% apoptotic cells). As shown in Figure 4F, consistent with the cell viability study, FLJ-siRNA-1 transfected cells exhibited a significant increase in sensitivity to apoptosis when treated with 5 µg/ml DDP, relative to negative control cells (A549∶32.47±3.21% apoptotic cells vs 15.87±1.56%, p<0.05; L9981∶42.45±2.56% apoptotic cells vs 21.54±2.45%, p<0.05).

### Gene Expression Profile of FLJ20420-silenced A549 Cells

The biological function of FLJ20420 was further investigated by analyzing the gene expression profile of A549 cells transfected with FLJ20420-siRNA. Microarray analysis revealed 655 upregulated genes, including 272 known genes (272/38,500 = 0.706%) and 384 unknown genes. A total of 619 downregulated genes were identified, including 233 known genes (233/38,500 = 0.605%) and 386 unknown genes, as shown in Table S1 and S2. Further analysis revealed that many of the differentially expressed genes were involved in cell signaling pathways, such as: apoptosis, calcium signaling pathway, cell cycle, cell junctions, cytokine-cytokine receptor interaction, ECM-receptor interaction, ErbB signaling pathway, glycolysis/gluconeogenesis, insulin signaling pathway, Jak-STAT signaling pathway, MAPK signaling pathway, p53 signaling pathway, TGF-beta signaling pathway, Toll-like receptor signaling pathway, ubiquitin-mediated proteolysis and Wnt signaling pathway (details given in Table S3).

**Figure 5:**
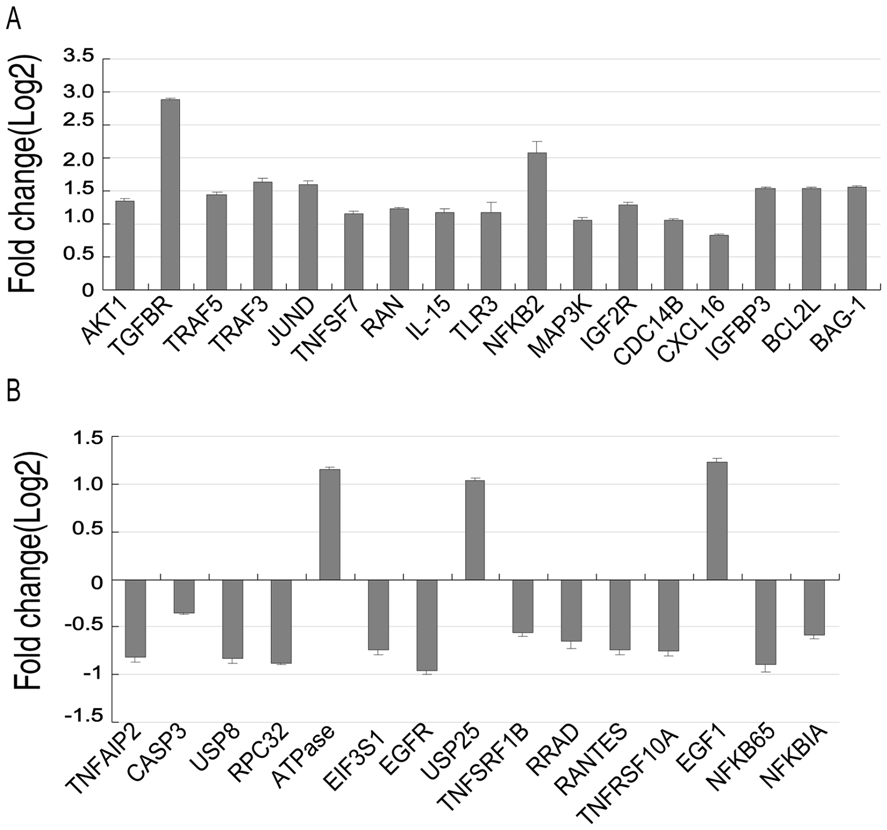
Real-time PCR analysis of gene expression in A549 cells transfected with FLJ20420 siRNA. Compared to control transfectants, a log_2_ signal ratio ≥ 1 or ≤ 1 was considered to be a significant change in gene expression. (A) The expression level changes of 17 genes shown to be upregulated by microarray. (B) Downregulated expression levels of 15 genes, as determined by microarray analysis. Among the 17 upregulated genes detected by microarray analysis, real-time PCR confirmed 15 of these genes to have significant transcriptional expression changes, while 2 did not show any significant change. Among the 15 downregulated genes detected by microarray analysis, real-time PCR confirmed 12 of these genes to have significant transcriptional expression changes, while 3 did not show any significant changes.

Real-time PCR was also used to verify the upregulated and downregulated genes that were identified by microarray analysis (total of 32 genes). As shown in Figure 5, the transcriptional expression of 15 genes including BAG-1, BCL 2L, IGFBP3, CDC14B, JUND, TRAF3, TGFBR, AKT1, TRAF5, NFKB2, TNFSF7, TLR3, MAP3K, IGF2R and RAN were significantly increased compared to control cells (Figure 5A). In contrast, gene expression levels of TNFAIP2, USP8, RPC32, EIF3S1, EGFR, TNFSF1B, RRAD, RANTES, TNFRSF10A, NFKB65 and NFKBIA were unmistakably decreased (Figure 5B). However, the altered expression of certain upregulated genes (i.e., CXC16, IL15) and downregulated genes (i.e., USP25, EGF1, ATPase and CASP3) were not observed by real-time PCR. Therefore, of the 32 analyzed genes, an 84.3% (27/32) concordance in expression was determined between the microarray and real-time PCR data.

### FLJ20420 and BAG-1 Expression in Human Lung Tumor tissues

**Figure 6:**
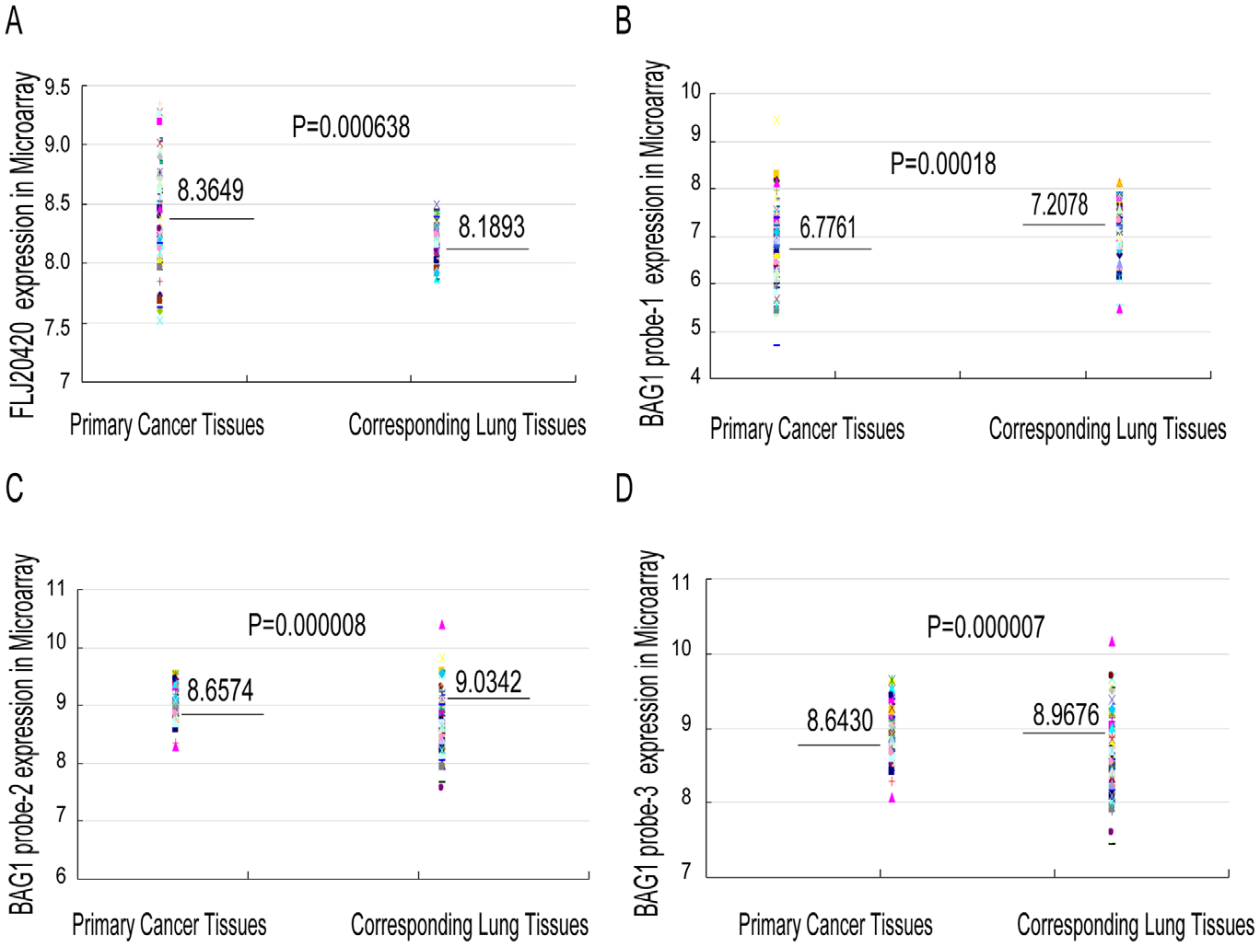
FLJ20420 and BAG-1 gene expression in lung tumor tissues. Microarray analysis of FLJ20420 (A) and BAG-1 in lung tumor tissues (B-D), relative to the paired normal lung tissues.

Next, we determined the expression of FLJ20420 and BAG-1 genes in primary lung cancer tissues and the corresponding paired normal controls. Microarray analysis was carried out in 72 paired lung tissue specimens, which included 29 adenocarcinomas and 43 squamous cell carcinomas. As shown in Figure 6A-D, expression of FLJ20420 mRNA was significantly higher in primary lung cancer tissues compared to the paired normal controls (mean: 8.3649 vs 8.1893, P = 0.0006). In contrast, lower expression levels of BAG-1 mRNA were observed in isolated primary lung tumor tissues compared to the paired normal controls (mean: BAG-1 probe 1∶6.7761 vs 7.2078, P = 0.00018; probe 2∶8.643 vs 8.9676, P = 8.64E-06; probe 3∶8.6574 vs 9.0345, P = 7.19E-07).

## Discussion

Although several proteins are known to regulate BAG-1 expression, (i.e., interleukin-2 (IL-2), interferons (IFNs) and granulocyte-macrophage colony-stimulating factor (GM-CSF)) none have been shown to bind directly to the BAG-1 promoter [14], [15], [16]. To understand the molecular regulation of BAG-1 expression in human cancer, we have identified a cDNA that encodes a novel BAG-1 transcription factor, termed FLJ20420, using a protein-DNA fragment interaction cloning technique. The predicted amino acid sequence of FLJ20420 has no clear similarity to any other known proteins. Therefore, FLJ20420 may represent a novel BAG-1-regulating gene.

FLJ20420 is an uncharacterized coiled-coil-helix-coiled-coil-helix domain-containing protein 3 (Chchd3) that possesses sequence similarity with mouse Chchd3 (90%; GI: 62510510), rat Chchd3 (84%; GI: 62646993) and chimpanzee Chchd3 (99%; GI: 55629442). In addition, comparison of the conserved domain of FLJ20420 to known proteins in GenBank, has identified significant homology with DUF737, a protein of unknown function. These proteins are part of a family with several uncharacterized mammalian proteins of unknown function. In 2005, by combined proteome analysis and *in situ* hybridization, Dreger reported that rat FLJ20420 mRNA exhibited a high level of expression in the majority of neurons of the dorsal root ganglion and the spinal cord, as well as in many types of rat brain cells [17]. Quantitative gene expression profiling in a neurodegenerative mouse model for Huntington’s disease did not find a significant difference in Chchd3 expression in R6/2 or wild-type mice [18]. Chen et al studied the differential gene expression of interstitial cells of Cajal in the murine small intestine and showed that Chchd3 expression is higher in the region of the myenteric plexus than in the deep muscular plexus [18]. FLJ20420 has also been found to be a mitochondrial structural protein, where its depletion promoted mitochondrial loss and autophagy [19]. In our experiments, we detected a low level of FLJ20420 expression in human brain, placenta, lung, liver, kidney, pancreas and cervix, and a very high level of expression in heart and skeletal muscle. These data are consistent with other reports regarding increased FLJ20420 expression levels in heart tissue [20]. FLJ20420 has been speculated to play a role in energy production and transcription/translation processes in heart muscle, since it is a part of the PKCε complexes that contain metabolism-, transcription-, and translation-related proteins [21] that are known to regulate cellular metabolism, protein expression and protein synthesis. Alternation between transcription factor activation and protein expression is necessary to protect the heart from ischemia [22].

BAG-1 is a multifunctional protein that interacts with a wide range of cellular targets including heat shock proteins and some nuclear hormone receptors. BAG-1 is also over-expressed in several human malignancies, especially in human breast cancer and cervical cancer. In this study, we demonstrated that the FLJ20420 protein specifically binds to the BAG-1 promoter and functions as a negative transcription factor for BAG-1. Co-transfection of the BAG-1 promoter with FLJ20420 leads to a decrease in *in vitro* promoter activity. This result was also confirmed by knockdown of endogenous FLJ20420 expression which leads to increased *in vivo* BAG-1 expression. We also observed a concordant decrease in BAG-1 expression and increase in FLJ20420 expression in lung cancer cell lines and the paired normal tissue controls. These results suggest that FLJ20420 functions as a negative regulator of BAG-1. BAG-1 can be expressed as four protein products that are generated by alternative translation initiation from a single transcript. Although it has been suggested that the four BAG-1 isoforms are translated by leaky *in vitro* scanning, other studies have proposed that an additional mechanism may be involved. Willis and co-workers have shown that the *in vivo* synthesis of BAG-1S is dependent upon the presence of an internal ribosome entry segment (IRES) in the 5′-UTR of BAG-1 mRNA [11]. In our studies, only the expression of BAG-1 isoforms, p50 and p33, were found to be increased in FLJ20420-siRNA-transfected A549 and L9981 cells, as shown by Western blot analysis. We did not observe any changes in the expression of BAG-1 p46 and p29 isoforms in FLJ20420-silenced cells. Translation of BAG-1 p50, which contains an SV40-like nuclear localization signal, is initiated at a non-canonical CUG codon. Furthermore, translation of BAG-1 p33 is dependent on the IRES in the 5′-UTR of BAG-1 mRNA. These data demonstrate that the expression of the four BAG-1 isoforms is regulated by multiple mechanisms including leaky scanning and IRES, which are both tissue-specific. Current studies are investigating the role of FLJ20420 in the production and function of different BAG-1 isoforms in various normal and cancer tissues.

The precise biological function of FLJ20420 still remains unclear. Considering the role of BAG-1 in apoptosis, we did not find any significant changes in cell cycle or cell viability in FLJ-20420-silenced A549 and L9981 cells. These data suggest that FLJ20420 alone may not regulate these events, and also imply that BAG-1 expression may be controlled by additional transcription factors. Furthermore, the role of FLJ20420 in tumor apoptosis was investigated by studying the effect of downregulated FLJ20420 in cisplatin-treated cancer cells. Unexpectedly, we found that the FLJ20420-silenced cells were more sensitive to cisplatin-induced apoptosis. One explanation for this discrepancy is that FLJ20420 may affect apoptosis through other apoptotic proteins, apart from BAG-1. Mitofilin, a mitochondrial inner membrane protein that controls crista morphology, may be an example of such a protein. Previous studies have shown that knockdown of mitofilin expression in HeLa cells leads to decreased cell proliferation and increased apoptosis [23]. Since the FLJ20420 protein is a known component of mitofilin complexes [24], it may also modulate cell apoptosis by interacting with these complexes. In addition, other apoptosis-controlling proteins may be involved in FLJ20420-regulated apoptosis. Our microarray studies demonstrated that siRNA-mediated FLJ20420 downregulation altered the expression of many apoptosis-related genes. For example, the expression levels of CASP7, MX1 and BOK were all observed to be upregulated, while the expression levels of OPA1, MCL1, APPBP2, TNFRSF10A, CFLAR and CASP3 were dramatically downregulated. Although the expression of many apoptosis-related genes changed after silencing of FLJ20420, flow cytometry analysis did not reveal a clear difference in the percentage of apoptotic cells between FLJ20420-siRNA and negative control-siRNA transfectants. However, FLJ20420-silenced cells were more sensitive to cisplatin-induced apoptosis. These data suggest that downregulation of FLJ20420 expression alone is not sufficient to induce apoptosis, but is sufficient to sensitize cells to apoptosis-inducing agents such as cisplatin.

Recent studies have demonstrated that Chchd3 is a novel PKA substrate in mitochondria [25]. Chchd3 contains an N-terminal myristylation site followed by a domain of unknown function (DUF) and a CHCH domain (coiled-helix-coiled-helix domain). The N-terminus of Chchd3 is highly conserved within vertebrates, and contains a well conserved PKA phosphorylation site at Thr10 or Ser10. Chchd3 is also known to localize to the matrix space in mitochondria. Mitochondrial proteomic analysis has shown that FLJ20420 is expressed in the mitochondria [26], while expression quantitative trait loci (eQTL) analysis of genome-wide gene expression profiling suggests that FLJ20420 may play a role in oxidative phosphorylation [27]. Other studies have reported that FLJ20420 is a lipid raft-associated protein that is involved in triggering the activation of T-cell antigen receptors [28]. Until now, the exact physiological role of FLJ20420 has remained largely unknown. In this study, we have clearly identified the role of FLJ20420 as a BAG-1 transcription factor, and also linked its expression to sensitivity to cisplatin-induced apoptosis in cancer cells. Knockdown of FLJ20420 expression significantly increased the expression of BCL-2L, IGFBP3, CDC14B, JUND, TRAF3, TGFBR, AKT1, TRAF5, NFKB2, TNFSF7, TLR3, MAP3K, IGF2R and RAN, but decreased the expression of TNFAIP2, USP8, RPC32, EIF3S1, EGFR, TNFSF1B, RRAD, RANTES, TNFRSF10A, NFKB65 and NFKBIA. Interestingly, many of these proteins are known to be involved in apoptosis. Nevertheless, ongoing studies in our laboratory are focused on determining whether FLJ20420 directly or indirectly regulates the expression of these genes. In addition, knockdown of FLJ20420 expression also significantly increased the expression of PRKCBP2, TGFBR1, TGFB2, CLCN4, TRAF3, MYLK and ACTA2, while decreasing the expression of SMAD5 and prot-LBC, resulting in an increased apoptotic potential in FLJ20420-silenced lung cancer cells. This observation, which is consistent with previous reports identifying FLJ20420 as a novel PKA substrate in mitochondria, also helped to connect FLJ20420 with the PKA signaling pathway [25]. Future studies in our laboratory will investigate the molecular mechanism that allows FLJ20420 to upregulate the expression of PRKCBP2, TGFBR1, and TGFB2 and to downregulate the expression of SMAD5 and prot-LBC.

In these studies, FLJ20420 was observed to be expressed at low levels in the majority of normal tissues. Interestingly, this is the first report demonstrating a significant increase in FLJ20420 expression in primary lung tumors, relative to paired normal lung tissues. In contrast, BAG-1 expression in primary lung tumor tissues was found to be significantly lower than in the paired normal lung tissues. We also demonstrated an inverse relationship between the expression of FLJ20420 and BAG-1 in lung tumor tissues. Due to the small number of tissue specimens and short follow-up time, we were unable to analyze the relationship between FLJ20420 expression and other clinical parameters such as stage, differentiation, response to treatment and survival. We previously reported that BAG-1 overexpression in a large number of lung cancers is linked to improved patient prognosis [29]. Future studies at our institution will determine whether increased FLJ20420 expression can also be found in the lung cancer patient population. Additionally, BAG-1 expression is also known to be regulated by other proteins such as IFNs, IL-2 and GM-CSF. It will be interesting to determine the expression of these cytokines in these paired lung cancer and normal tissue samples, to correlate their expression to FLJ20420 and BAG-1 expression, and also to analyze their expression in a large number of lung cancer patient samples. A deeper understanding of FLJ20420 in lung cancer and other malignancies is important in the development of BAG-1-targeted therapy to improve the prognosis and treatment of patients with cancer.

In summary, this is the first identification and characterization of a negative BAG-1 regulating transcription factor. Our studies demonstrated that FLJ20420 specifically binds to the BAG-1 promoter and decreases its expression. Our data also suggest that FLJ20420 plays an important role in apoptosis and oncogenesis in lung cancer. Current studies in our laboratory seek to unravel the mechanisms by which FLJ20420 regulates the expression of BAG-1 and its isoforms, as well as additional apoptotic-related proteins in lung cancer and other malignancies.

## Materials and Methods

### Screening of the Human HeLa 5′-stretch Plus cDNA Library, λTripIEx, with the BAG-1 Promoter

BAG-1 promoter probes of different sizes have been previously reported [13]. The Human HeLa 5′-stretch plus cDNA library, λTripIEx, was purchased from Clontech. Screening of the cDNA expression library was performed using the Southwestern blot method, previously described by Vinson et al [30]. Positive plaques were identified and re-screened until they were homogeneously positive. We then converted homogeneously positive plaques from the λTripIEx to pTripIEx according to the manufacturer’s instructions. The positive plaque was then sequenced. Homology searches were performed using BLAST (Basic Local Alignment Search Tool) from the National Center for Biotechnology Information (NCBI) available at http://www.ncbi.nlm.gov.

### Cell Culture, Transfection, Immunofluorescence and Gene Silencing

Human cervical cancer cell line HeLa, NIH/3T3 (mouse embryonic fibroblast cell line), A549 (human lung adenocarcinoma cell line), NCI-H460 (human large cell lung cancer), NCI-H446 and SHP-77 (human small cell lung carcinoma cell lines) were from the American Tissue Culture Collection (ATCC). 95 C (human lung carcinoma cell line), 95 D (human lung carcinoma cell line) were purchased from the Chinese Academy of Sciences Committee Cell Culture Collection. SPCA-1,YTMLC-9, GLC-82, LTEP-α-2 were produced in our institution. Human large cell lung cancer cell lines L9981 and NL9980 were established by our institute [31]. HeLa and NIH/3T3 were maintained in DMEM, and the rest of cell lines were maintained in the RPMI 1640 containing 10% fetal bovine serum (GIBCO) at 37°C with 5% CO_2._ DNA transfection was performed using the Lipofectamine^TM^ 2000 reagent (Invitrogen, CA), according to the manufacturer’s instructions.

Immunofluorescence studies were conducted by seeding 1x10^4^ cells/well on 4-well BD Falcon™ Culture Slides (BD Biosciences) as previously described by Lu et al [32]. The slides were then incubated overnight at 37°C with 5% CO_2_. Transfections were carried out by mixing 0.6 µg pEF1-HisC-FLJ20420 with the Lipofectamine^TM^ 2000 reagent into Hela and NIH/2T3 cells. The transfection mixture was added to the cell monolayer and the cells were incubated for an additional 48 h. The washed cells were incubated with the monoclonal anti-Xpress^TM^ antibody (Invitrogen, CA) at a dilution of 1∶500, for 2 h at room temperature and then with the secondary antibody, Cy^TM^2-conjugated AffiniPure goat anti-mouse IgG (H+L) (Jackson ImmunoResearch Laboratories, green fluorescence), at a dilution of 1∶200, for another 2 h at room temperature. The cytoskeleton and nuclei were visualized by staining with phalloidin (Sigma, red fluorescence) and DAPI (Sigma, blue fluorescence) at dilutions of 1∶500 and 1∶5000, respectively. The same experiment was performed in A549 cells with the monoclonal anti-FLJ20420 antibody, purchased from the Santa Cruz Biotechnology.

Knockdown of FLJ20420 protein expression was performed using the FJL20420 specific siRNAs (Cat#: siB0892790128 and 2005815122147, Guangzhou Ribobio CO., LTD, China). The FLJ 20420 siRNA sequences were as follows: FLJ-siRNA-1: sense: 5′-GAGCAAGCCAAG AAAGAAU dTdT-3′, anti-sense: 5′-dTdTCUCGUUCGGUUCUUUC UUA-3′; FLJ-siRNA-2: sense: 5′-GCGGUAUUCUGGUGCU UAU dTdT-3′ and anti-sense: 3′-dTdTCGCCAUAAG ACCACGAAUA-5′. Scrambled siRNA, a functional non-targeting siRNA provided by the same company, was used as a negative control. Briefly, in a 6-well plate, 2×10^5^ A549 or L9981 cells/well were transfected with 100 pmol of FJL20420 siRNA or control siRNA using Lipofectamine^TM^ 2000 (Invitrogen). After incubation at 37°C with 5% CO_2_ for 48 h, the cells were collected and prepared for analysis by real-time PCR and Western blotting.

### Construction of Recombinant Plasmids

Unless otherwise stated, all plasmids were constructed by inserting the sequence shown below, between the EcoRI and XhoI sites. The primers used were: forward 5′-gc gaa ttc atg ggt ggg acc acc agc acc cg-3′; reverse 5′-gtc ctc gag tta tcc tcc ctt ctc aag cat-3′. PCR amplification of the positive pTripIEx-plaques was used to generate the regions inserted into the pcDNA3.1-FLJ20420, pcDNA3.1/HisC-FLJ20420, pEF1-HisC-FLJ20420 and pGEX-4T-1-FLJ20420 constructs. All constructed plasmids were confirmed by DNA sequencing.

### FLJ20420 Protein Induction and in vitro Translation

The pGEX-4T-1-FLJ20420 construct was grown in BL21 cells, and induced with 0.1 mM IPTG at 18°C overnight. The GST-FLJ20420 fusion protein expressed was purified using the MicroSpin GST purification module (Amersham). The TNT Quick Coupled Transcription/Translation kit (Promega) was used to translate the HisC-FLJ20420 fusion protein from the vector pcDNA3.1/HisC-FLJ20420, which was then subjected to Western blot analysis with monoclonal anti-His antibody (Invitrogen).

### Electrophoretic Mobility Shift Assays (EMSA)

EMSAs were carried out according to the protocol described by Chen et al [33]. Briefly, the full-length BAG-1 promoter was first separated by PCR into several DNA fragments of approximately150-200 bp in length (primer sequences are available upon request). The positive DNA fragment was then separated into smaller fragments until 30-50 bp oligos were obtained. Each oligonucleotide possessed 10 bp of overlapping sequence. Unlabeled specific and non-specific oligonucleotides were included at concentrations of 10, 100, and 1000 ng/40 µl.

### Chromatin Immunoprecipitation (ChIP) assay

The ChIP assay was performed using the EZ-Chip Chromatin Immunoprecipitation kit from MILLIPORE according to the manufacturer’s instructions. Briefly, crosslinking of protein and DNA was achieved by treating the Hela cells with 1% formaldehyde and then with lysis buffer containing protease inhibitor cocktail. Sonication to shear DNA was done using the VCX130 (Sonics&Materials, Newtown, USA) at 30% amplitude, 10-s pulses, 40-s rest, 12 times. Immunoprecipitation of crosslinked protein/DNA was performed with the polyclonal anti-human FLJ20420 antibody (SIGMA-ALDRICH, St. Louis, USA) at 4°C overnight. The protein/DNA complexes were eluted to uncouple the proten/DNA complexes to free the DNA, which was purified by spin columns. The full length BAG-1 promoter was separated as a 200-bp DNA fragment. The positive PCR primers that amplified −513 ∼−338 bp (175 bp DNA fragment) upstream of the BAG start codon are as follows: BGP1-4F: TCAGAGGTCCTGAG CCTACT, BGP1-4R: GGAGTGACTCTGCTTCCGTTT.

### Luciferase Assays

The pGL3-BGP1 BAG-1 full-length promoter plasmid was used as previously described [13]. The pGL3-Control (Promega) was used as a positive control. A549 and L9981 cells were co-transfected in 6-well plates (5 × 10^4^ cells/ml) with 0.8 µg luciferase reporter, 0.4 µg pcDNA3.1-FLJ20420, and 0.4 µg control vector, pcDNA3.1 (+). Transfection efficiency was determined by co-transfection of the pRL-CMV vector (0.02 µg) which encodes the Renilla luciferase gene. Cells were harvested after 48 h of incubation and luciferase activity measured using the Dual-luciferase assay reagent (Promega).

### Patients and Tissue Specimens

We studied the frozen specimens of lung cancer tissues and their paired normal lung tissues from over 2 cm away from the tumor sites. Seventy-two patients who underwent surgical resection for non-small-cell lung cancer (NSCLC) at the Tianjin Medical University General Hospital between December 2006 and August 2008 were included. As shown in Table 1, of these 72 patients, 29 specimens were adenocarcinomas, and 43 specimens were squamous-cell carcinomas. All of the specimens were diagnosed on the basis of histologic and immunohistologic criteria, according to the current World Health Organization (WHO) classification. The specimens were stored in liquid nitrogen. This study was approved by the Review Board of Tianjin Medical University General Hospital.

**Table 1:** Patient characteristics.

Type	Sub-type	N (%)
Gender	Male	55(76.4%)
	Female	17(23.6%)
Age	> = 61	37(51.4%)
	<61	35(48.6%)
Differentiation	low	21(29.2%)
	medium	31(43.1%)
	high	16(22.2%)
	mixed	4(5.6%)
Histology	adenocarcinoma	29(40.3%)
	Squamous cell carcinoma	43(59.7%)
Stage	I	14(19.4%)
	II	10(13.9%)
	III	43(59.7%)
	IV	5(6.94%)
Metastatic status	none	20(27.8%)
	Yes	52(72.2%)
Smoking status	Never	20(27.8%)
	Smoker	52(72.2%)

### RNA Isolation and Northern Blotting

Total RNA was extracted from 10^7^ cells or 100 mg tissue using the Trizol reagent (Invitrogen), according to the manufacturer’s instructions, and quantified by UV spectrophotometry (Beckman Coulter). Northern blot analysis was performed as previously described [14] using ^32^P-dCTP-labeled human FLJ20420 cDNA as a probe. Human MTN^TM^ Blot was purchased from Clontech (Mountain View, CA). Human β-actin cDNA was used as an internal control.

### Reverse Transcription and Real-time PCR

Total RNA (2 µg) was reverse-transcribed using the M- MLV Reverse Transcriptase kit (Promega), according to the manufacturer’s protocol. Twenty nanograms of the resultant cDNA were mixed with the ABI SYBR Green Master Mix (ABI) as well as the gene primers, and then amplified with the ABI7500 Real-time PCR System, according to the manufacturer’s protocol. Each experiment was run in triplicate. The results were analyzed by comparison of the 2^–averageΔΔCT^. The primers used for real-time PCR are as follows: FLJ20420 forward: 5′-ACGAGAATGAGAACATCACCG-3′, FLJ20420 reverse: 5′-CAGCTCCTCAGCTACTCTTC-3′; BAG-1 forward: 5′-GGCATTCCTAGCCG AGTGTG-3′, BAG-1 reverse: 5′-CCAGGGCAAAGTTTGTAGACTG-3′. PGK-1 was used as an internal control, as previously described [34].

### Microarray Assay

The Human Genome U133 Plus 2.0 microarray, with 54,000 probe sets was purchased from the Affymetrix (Lot #: 4032359). Total RNA was extracted with the Trizol reagent (Invitrogen) from the FLJ20420-siRNA transfected cells, negative control cells, or patients’ lung tissue samples (i.e., primary lung tumor tissues and the corresponding paired lung tissues). The RNA extracted was purified using the Oligotex mRNA Midi kit (Qiagen). Microarray assay was previously described [31]. In the screening process, the genes were considered to be differentially expressed if the log_2_ signal ratio was ≥1 (upregulated) or ≤1 (downregulated) [35].

### Western Blot Analysis

Cells were homogenized in RIPA buffer (50 mM Tris-HCl, pH 7.4; 150 mM NaCl; 1% Nonidet P-40; 0.5% sodium deoxycholate; 0.1% SDS; 1 mM EDTA; 1 mM PMSF; 1 mg/ml aprotinin). The protein concentrations were measured using the bicinchoninic acid (BCA) protein assay kit (Pierce). Twenty micrograms of whole-cell lysate were separated on a 10–15% SDS-PAGE gel, transferred to a nitrocellulose (NC) membrane (Amersham Biosciences) and immunoblotted with a monoclonal anti-β-actin antibody purchased from Sigma-Aldrich and monoclonal anti-BAG-1 antibody obtained from the Santa Cruz Biotechnology.

### MTT Assay

After transfection with NEG or FLJ20420 siRNA for 48 h in 6-well plates, the cells were trypsinized and seeded in 96-well plates at 1×10^4^/well. They were then exposed to an increasing concentration of cisplatin ranging from 0 to 40 µg/ml for 24 h. Twenty microliters of MTT solution was added to each well, and the cells were incubated for 4 h at 37°C to allow MTT to be converted to formazan crystals by reacting with metabolically active cells. Subsequently the formazan crystals were solubilized by 150 µl of DMSO. The absorbance of each well was measured with a microplate reader at 490 nm (A490).

### Flow Cytometry Analysis of Cell Cycle and Apoptosis

Flow cytometry was used to determine the effect of FLJ20420 on the cell cycle. First, 2×10^5^ cells/well were seeded in 6-well plates and incubated for 12 h. Cell synchronization was achieved by starvation in serum-free RPMI-1640 medium for 24 h. Afterwards, the cells were then transfected with the indicated siRNA and incubated for 48 h. The cells were then harvested and fixed in ice-cold 70% ethanol overnight. The cells were treated with DNase-free ribonuclease (TAKARA), stained with propidium iodide (PI) (Sigma-Aldrich), and then subjected to a FACSAria^TM^ flow cytometry (Becton Dickinson). The data were analyzed with the ModFit LT software.

Detection of annexin V binding to apoptotic cells was used to determine the effect of FLJ20420 siRNA on cell apoptosis. First, 2×10^5^ cells/well were seeded in 6-well plates and transfected with either FLJ20420-siRNA or control siRNA. The cells were then treated with 5 µg/ml cisplatin for 24 h, stained using the Annexin V-FITC Apoptosis Analysis kit (Pharmingen) and subjected to FACStar plus flow cytometry (Becton-Dickenson) to sort out the annexin V-FITC-stained apoptotic cells. The data were analyzed by first determining the percentage of apoptotic cells in the treated cell population and then subtracting the percentage of apoptotic cells in the untreated control cell population.

### Statistical Analysis

Data are presented as the mean±SD. The t-test was used to analyze the differences between the negative control and the FLJ20420-siRNA-transfected groups, as well as the differences between the lung cancer tissues and the paired normal lung tissue controls. All of the tests were two-sided. A P value of <0.05 was considered statistically significant.

## Supporting Information

Figure S1 **Sequence analysis of positive cDNA clone and the deduced amino acid sequence.** Numbers indicate nucleotide positions.
(TIF)

Figure S2 **Expression of FLJ20420 fusion protein.** (a) GST-FLJ20420 fusion protein was induced in BL21 cells with 0.1 mM IPTG at 18°C overnight. Lane M contains SeeBlue plus2 standard protein marker (Invitrogen); protein extractions for Lanes 1 and 2 were from uninduced or induced pGEX-4T-FLJ20420-transformed BL21 cells, respectively; lane V consists of the positive control for the GST vector. (b) The His-FLJ20420 fusion protein was translated *in vitro* using the TNT Quick Translation kit (Promega) and identified by Western blotting with an anti-His antibody.
(TIF)

Table S1 **The upregulated genes in A549-FLJ-siRNA cells.**
(XLS)

Table S2 **The downregulated genes in A549-FLJ-siRNA cells.**
(XLS)

Table S3 **The differentially expressed genes involved in cell signaling pathways in FLJ20420-silenced A549 cells**.
(DOC)
