# Supplementary figures and images for: Cloning and Functional Analysis of FLJ20420: A Novel Transcription Factor for the BAG-1 Promoter
Source: PLoS One. 2012 May 2;7(5):e34832. doi: 10.1371/journal.pone.0034832 (PMC3342300; doi:10.1371/journal.pone.0034832)

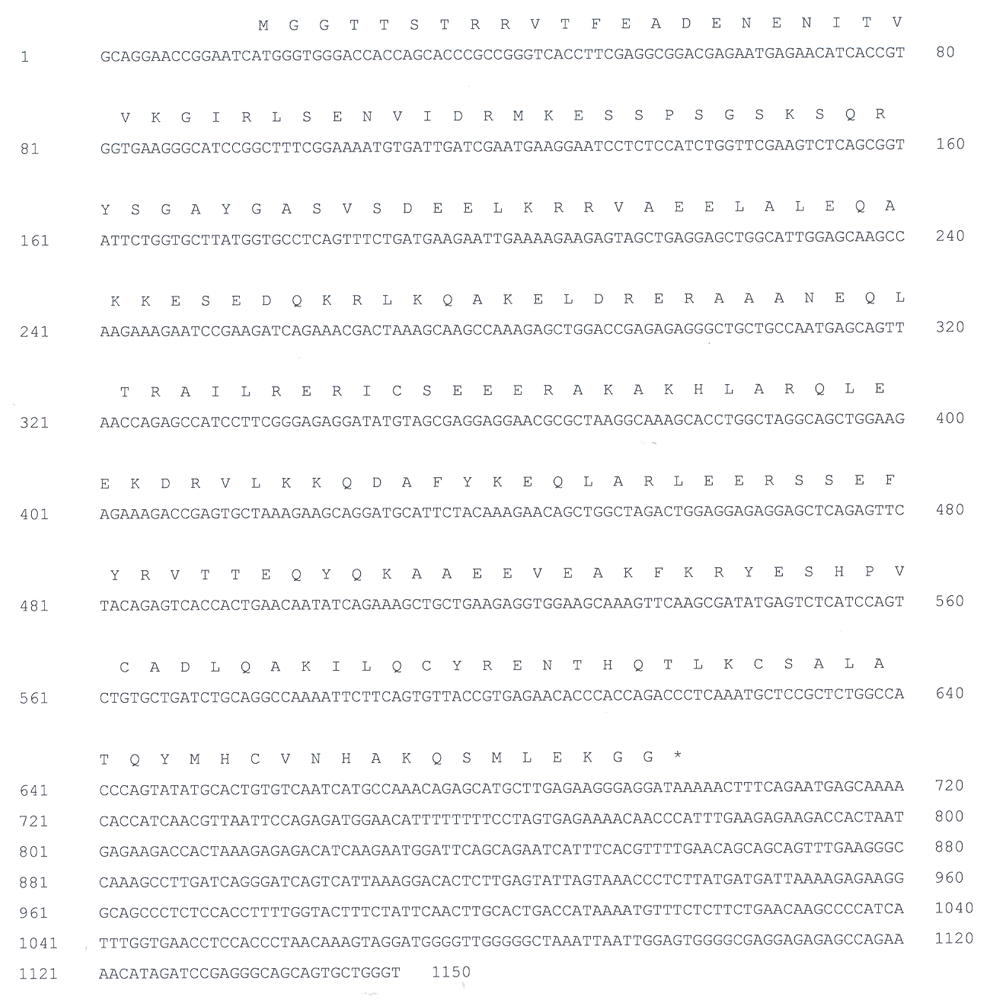

Supplement: Figure S1 — Sequence analysis of positive cDNA clone and the deduced amino acid sequence. Numbers indicate nucleotide positions. (TIF) [file pone.0034832.s001.tif]

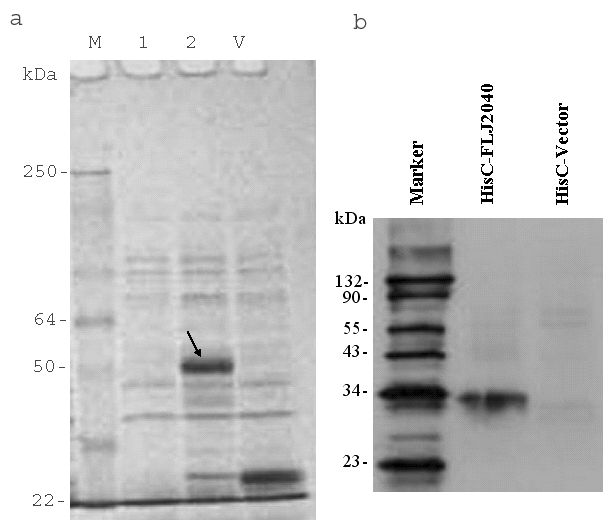

Supplement: Figure S2 — Expression of FLJ20420 fusion protein. (a) GST-FLJ20420 fusion protein was induced in BL21 cells with 0.1 mM IPTG at 18°C overnight. Lane M contains SeeBlue plus2 standard protein marker (Invitrogen); protein extractions for Lanes 1 and 2 were from uninduced or induced pGEX-4T-FLJ20420-transformed BL21 cells, respectively; lane V consists of the positive control for the GST vector. (b) The His-FLJ20420 fusion protein was translated in vitro using the TNT Quick Translation kit (Promega) and identified by Western blotting with an anti-His antibody. (TIF) [file pone.0034832.s002.tif]
