# Supplementary material for: Cloning and Functional Analysis of FLJ20420: A Novel Transcription Factor for the BAG-1 Promoter
Source: PLoS One. 2012 May 2;7(5):e34832. doi: 10.1371/journal.pone.0034832 (PMC3342300; doi:10.1371/journal.pone.0034832)
Supplement: Table S3 — The differentially expressed genes involved in cell signaling pathways in FLJ20420-silenced A549 cells. (DOC) [file pone.0034832.s005.doc]

Table S3: The differentially expressed genes involved in cell signaling pathways in FLJ20420-silenced A549 cells

| Cell signaling pathways | Genes with altered expression |
| --- | --- |
| Apoptosis | AKT1, BCL2L1, [CASP3](http://www.genome.ad.jp/dbget-bin/show_pathway?MAP04210+3.4.22.56), [CASP7](http://www.genome.ad.jp/dbget-bin/show_pathway?MAP04210+3.4.22.-), [CFLAR](http://www.genome.ad.jp/kegg/pathway/hsa/hsa04210.html), [CYCS](http://www.genome.ad.jp/kegg/pathway/hsa/hsa04210.html), [IL1RAP](http://www.genome.ad.jp/kegg/pathway/hsa/hsa04210.html), [PIK3CB](http://www.genome.ad.jp/dbget-bin/show_pathway?MAP04210+2.7.1.153), [TNFRSF10A](http://www.genome.ad.jp/kegg/pathway/hsa/hsa04210.html) |
| Calcium signaling pathway | [ADRB2](http://www.genome.ad.jp/kegg/pathway/hsa/hsa04020.html), [CAMK2D](http://www.genome.ad.jp/dbget-bin/show_pathway?MAP04020+2.7.11.17), [ERBB2](http://www.genome.ad.jp/dbget-bin/show_pathway?MAP04020+2.7.10.1), [MYLK](http://www.genome.ad.jp/dbget-bin/show_pathway?MAP04020+2.7.11.18), [OXTR](http://www.genome.ad.jp/kegg/pathway/hsa/hsa04020.html), [PLCB4](http://www.genome.ad.jp/dbget-bin/show_pathway?MAP04020+3.1.4.11), [PTK2B](http://www.genome.ad.jp/dbget-bin/show_pathway?MAP04020+2.7.10.2), [VDAC3](http://www.genome.ad.jp/kegg/pathway/hsa/hsa04020.html) |
| Cell cycle | [CCNE2](http://www.genome.ad.jp/kegg/pathway/hsa/hsa04110.html), [CDC2](http://www.genome.ad.jp/dbget-bin/show_pathway?MAP04110+2.7.11.22), [CDK6](http://www.genome.ad.jp/dbget-bin/show_pathway?MAP04110+2.7.11.22), [MAD1L1](http://www.genome.ad.jp/kegg/pathway/hsa/hsa04110.html), [TGFB2](http://www.genome.ad.jp/kegg/pathway/hsa/hsa04110.html)), |
| Cell junctions | [COL5A1](http://www.genome.ad.jp/kegg/pathway/hsa/hsa01430.html), [GJA4](http://www.genome.ad.jp/kegg/pathway/hsa/hsa01430.html), [KRT8](http://www.genome.ad.jp/kegg/pathway/hsa/hsa01430.html), [SPP1](http://www.genome.ad.jp/kegg/pathway/hsa/hsa01430.html), [THBS1](http://www.genome.ad.jp/kegg/pathway/hsa/hsa01430.html), [VTN](http://www.genome.ad.jp/kegg/pathway/hsa/hsa01430.html) |
| Cytokine-cytokine receptor interaction | [ACVR2A](http://www.genome.ad.jp/dbget-bin/show_pathway?MAP04060+2.7.11.30), [CSF2](http://www.genome.ad.jp/kegg/pathway/hsa/hsa04060.html), [CXCL2](http://www.genome.ad.jp/kegg/pathway/hsa/hsa04060.html), [CXCL6](http://www.genome.ad.jp/kegg/pathway/hsa/hsa04060.html), [IFNAR1](http://www.genome.ad.jp/kegg/pathway/hsa/hsa04060.html), [IL1RAP](http://www.genome.ad.jp/kegg/pathway/hsa/hsa04060.html), [IL6](http://www.genome.ad.jp/kegg/pathway/hsa/hsa04060.html), [IL6R](http://www.genome.ad.jp/kegg/pathway/hsa/hsa04060.html), [IL6ST](http://www.genome.ad.jp/kegg/pathway/hsa/hsa04060.html), [INHBC](http://www.genome.ad.jp/kegg/pathway/hsa/hsa04060.html), [LEPR](http://www.genome.ad.jp/kegg/pathway/hsa/hsa04060.html), [PDGFA](http://www.genome.ad.jp/kegg/pathway/hsa/hsa04060.html), [TGFB2](http://www.genome.ad.jp/kegg/pathway/hsa/hsa04060.html), [TGFBR1](http://www.genome.ad.jp/dbget-bin/show_pathway?MAP04060+2.7.11.30), [TNFRSF10A](http://www.genome.ad.jp/kegg/pathway/hsa/hsa04060.html) |
| ECM-receptor interaction | [COL5A1](http://www.genome.ad.jp/kegg/pathway/hsa/hsa04512.html), [ITGA11](http://www.genome.ad.jp/kegg/pathway/hsa/hsa04512.html), [ITGA2](http://www.genome.ad.jp/kegg/pathway/hsa/hsa04512.html), [SPP1](http://www.genome.ad.jp/kegg/pathway/hsa/hsa04512.html), [THBS1](http://www.genome.ad.jp/kegg/pathway/hsa/hsa04512.html), [VTN](http://www.genome.ad.jp/kegg/pathway/hsa/hsa04512.html) |
| ErbB signaling pathway | [AKT1](http://www.genome.ad.jp/dbget-bin/show_pathway?MAP04012+2.7.11.1), [CAMK2D](http://www.genome.ad.jp/dbget-bin/show_pathway?MAP04012+2.7.11.17), [CBLB](http://www.genome.ad.jp/dbget-bin/show_pathway?MAP04012+6.3.2.19), [ERBB2](http://www.genome.ad.jp/dbget-bin/show_pathway?MAP04012+2.7.10.1), [GAB1](http://www.genome.ad.jp/kegg/pathway/hsa/hsa04012.html), [PIK3CB](http://www.genome.ad.jp/dbget-bin/show_pathway?MAP04012+2.7.1.153), [RPS6KB1](http://www.genome.ad.jp/dbget-bin/show_pathway?MAP04012+2.7.11.1) |
| Glycolysis / gluconeogenesis | [ADH6](http://www.genome.ad.jp/dbget-bin/show_pathway?MAP00010+1.1.1.1), [ALDH3B1](http://www.genome.ad.jp/dbget-bin/show_pathway?MAP00010+1.2.1.5), [HK2](http://www.genome.ad.jp/dbget-bin/show_pathway?MAP00010+2.7.1.1), [PFKL](http://www.genome.ad.jp/dbget-bin/show_pathway?MAP00010+2.7.1.11), [PGK1](http://www.genome.ad.jp/dbget-bin/show_pathway?MAP00010+2.7.2.3) |
| Insulin signaling pathway | [AKT1](http://www.genome.ad.jp/dbget-bin/show_pathway?MAP04910+2.7.11.1), [CBLB](http://www.genome.ad.jp/dbget-bin/show_pathway?MAP04910+6.3.2.19), [MKNK2](http://www.genome.ad.jp/dbget-bin/show_pathway?MAP04910+2.7.11.1), [PDE3A](http://www.genome.ad.jp/dbget-bin/show_pathway?MAP04910+3.1.4.17), [PFKL](http://www.genome.ad.jp/dbget-bin/show_pathway?MAP04910+2.7.1.11), [PIK3CB](http://www.genome.ad.jp/dbget-bin/show_pathway?MAP04910+2.7.1.153), [PPP1R3C](http://www.genome.ad.jp/kegg/pathway/hsa/hsa04910.html), [PTPRF](http://www.genome.ad.jp/dbget-bin/show_pathway?MAP04910+3.1.3.48), [RAPGEF1](http://www.genome.ad.jp/kegg/pathway/hsa/hsa04910.html), [RHEB](http://www.genome.ad.jp/kegg/pathway/hsa/hsa04910.html), [RHOQ](http://www.genome.ad.jp/kegg/pathway/hsa/hsa04910.html), [RPS6KB1](http://www.genome.ad.jp/dbget-bin/show_pathway?MAP04910+2.7.11.1) |
| Jak-STAT signaling pathway | [AKT1](http://www.genome.ad.jp/dbget-bin/show_pathway?MAP04630+2.7.11.1), [BCL2L1](http://www.genome.ad.jp/kegg/pathway/hsa/hsa04630.html), [CBLB](http://www.genome.ad.jp/dbget-bin/show_pathway?MAP04630+6.3.2.19), [CSF2](http://www.genome.ad.jp/kegg/pathway/hsa/hsa04630.html), [IFNAR1](http://www.genome.ad.jp/kegg/pathway/hsa/hsa04630.html), [IL6](http://www.genome.ad.jp/kegg/pathway/hsa/hsa04630.html), [IL6R](http://www.genome.ad.jp/kegg/pathway/hsa/hsa04630.html), [IL6ST](http://www.genome.ad.jp/kegg/pathway/hsa/hsa04630.html), [JAK3](http://www.genome.ad.jp/dbget-bin/show_pathway?MAP04630+2.7.10.2), [LEPR](http://www.genome.ad.jp/kegg/pathway/hsa/hsa04630.html), [PIAS1](http://www.genome.ad.jp/kegg/pathway/hsa/hsa04630.html), [PIK3CB](http://www.genome.ad.jp/dbget-bin/show_pathway?MAP04630+2.7.1.153) |
| MAPK signaling pathway | [AKT1](http://www.genome.ad.jp/dbget-bin/show_pathway?MAP04010+2.7.11.1), [BDNF](http://www.genome.ad.jp/kegg/pathway/hsa/hsa04010.html), [CASP3](http://www.genome.ad.jp/dbget-bin/show_pathway?MAP04010+3.4.22.56), [DUSP3](http://www.genome.ad.jp/dbget-bin/show_pathway?MAP04010+3.1.3.16), [FGF2](http://www.genome.ad.jp/kegg/pathway/hsa/hsa04010.html), [FGFR2](http://www.genome.ad.jp/dbget-bin/show_pathway?MAP04010+2.7.10.1), [JUND](http://www.genome.ad.jp/kegg/pathway/hsa/hsa04010.html), [MAP3K8](http://www.genome.ad.jp/dbget-bin/show_pathway?MAP04010+2.7.11.25), [MKNK2](http://www.genome.ad.jp/dbget-bin/show_pathway?MAP04010+2.7.11.1), [MRAS](http://www.genome.ad.jp/kegg/pathway/hsa/hsa04010.html), [PDGFA](http://www.genome.ad.jp/kegg/pathway/hsa/hsa04010.html), [PLA2G12A](http://www.genome.ad.jp/dbget-bin/show_pathway?MAP04010+3.1.1.4), [PPM1A](http://www.genome.ad.jp/dbget-bin/show_pathway?MAP04010+3.1.3.16), [RASGRP1](http://www.genome.ad.jp/kegg/pathway/hsa/hsa04010.html), [STK4](http://www.genome.ad.jp/dbget-bin/show_pathway?MAP04010+2.7.11.6), [TAOK1](http://www.genome.ad.jp/dbget-bin/show_pathway?MAP04010+2.7.11.1), [TGFB2](http://www.genome.ad.jp/kegg/pathway/hsa/hsa04010.html), [TGFBR1](http://www.genome.ad.jp/dbget-bin/show_pathway?MAP04010+2.7.11.30) |
| p53 signaling pathway | [CASP3](http://www.genome.ad.jp/dbget-bin/show_pathway?MAP04115+3.4.22.56), [CCNE2](http://www.genome.ad.jp/kegg/pathway/hsa/hsa04115.html), [CDC2](http://www.genome.ad.jp/dbget-bin/show_pathway?MAP04115+2.7.11.22), [CDK6](http://www.genome.ad.jp/dbget-bin/show_pathway?MAP04115+2.7.11.22), [CYCS](http://www.genome.ad.jp/kegg/pathway/hsa/hsa04115.html), [IGFBP3](http://www.genome.ad.jp/kegg/pathway/hsa/hsa04115.html), [PERP](http://www.genome.ad.jp/kegg/pathway/hsa/hsa04115.html), [PTEN](http://www.genome.ad.jp/dbget-bin/show_pathway?MAP04115+3.1.3.67), [THBS1](http://www.genome.ad.jp/kegg/pathway/hsa/hsa04115.html) |
| TGF-beta signaling pathway | [ACVR2A](http://www.genome.ad.jp/dbget-bin/show_pathway?MAP04350+2.7.11.30), [ID2](http://www.genome.ad.jp/kegg/pathway/hsa/hsa04350.html), [INHBC](http://www.genome.ad.jp/kegg/pathway/hsa/hsa04350.html), [RPS6KB1](http://www.genome.ad.jp/dbget-bin/show_pathway?MAP04350+2.7.11.1), [SMAD5](http://www.genome.ad.jp/kegg/pathway/hsa/hsa04350.html), [TGFB2](http://www.genome.ad.jp/kegg/pathway/hsa/hsa04350.html), [TGFBR1](http://www.genome.ad.jp/dbget-bin/show_pathway?MAP04350+2.7.11.30), [THBS1](http://www.genome.ad.jp/kegg/pathway/hsa/hsa04350.html) |
| Toll-like receptor signaling pathway | [AKT1](http://www.genome.ad.jp/dbget-bin/show_pathway?MAP04620+2.7.11.1), [IFNAR1](http://www.genome.ad.jp/kegg/pathway/hsa/hsa04620.html), [IL6](http://www.genome.ad.jp/kegg/pathway/hsa/hsa04620.html), [IRF5](http://www.genome.ad.jp/kegg/pathway/hsa/hsa04620.html), [MAP3K8](http://www.genome.ad.jp/dbget-bin/show_pathway?MAP04620+2.7.11.25), [PIK3CB](http://www.genome.ad.jp/dbget-bin/show_pathway?MAP04620+2.7.1.153), [SPP1](http://www.genome.ad.jp/kegg/pathway/hsa/hsa04620.html), [TLR1](http://www.genome.ad.jp/kegg/pathway/hsa/hsa04620.html), [TRAF3](http://www.genome.ad.jp/kegg/pathway/hsa/hsa04620.html) |
| Ubiquitin-mediated proteolysis | [CBLB](http://www.genome.ad.jp/dbget-bin/show_pathway?MAP04120+6.3.2.19), [CUL4A](http://www.genome.ad.jp/kegg/pathway/hsa/hsa04120.html), [FBXO2](http://www.genome.ad.jp/kegg/pathway/hsa/hsa04120.html), [MID1](http://www.genome.ad.jp/dbget-bin/show_pathway?MAP04120+6.3.2.19), [PIAS1](http://www.genome.ad.jp/kegg/pathway/hsa/hsa04120.html), [UBE2J1](http://www.genome.ad.jp/dbget-bin/show_pathway?MAP04120+6.3.2.19), [UBE2Q1](http://www.genome.ad.jp/dbget-bin/show_pathway?MAP04120+6.3.2.19) |
| Wnt signaling pathway | [CAMK2D](http://www.genome.ad.jp/dbget-bin/show_pathway?MAP04310+2.7.11.17), [PLCB4](http://www.genome.ad.jp/dbget-bin/show_pathway?MAP04310+3.1.4.11), [PPP2R5E](http://www.genome.ad.jp/kegg/pathway/hsa/hsa04310.html), [TCF7L1](http://www.genome.ad.jp/kegg/pathway/hsa/hsa04310.html), [VANGL1](http://www.genome.ad.jp/kegg/pathway/hsa/hsa04310.html), [WNT6](http://www.genome.ad.jp/kegg/pathway/hsa/hsa04310.html) |
